# The Interaction Between Physical and Psychosocial Stressors

**DOI:** 10.3389/fnbeh.2020.00063

**Published:** 2020-05-14

**Authors:** Esraa S. Abdelall, Zoe Eagle, Tor Finseth, Ahmad A. Mumani, Zhonglun Wang, Michael C. Dorneich, Richard T. Stone

**Affiliations:** ^1^Department of Industrial and Manufacturing Systems Engineering, Iowa State University, Ames, IA, United States; ^2^Industrial Engineering Department, Jordan University of Science and Technology, Irbid, Jordan; ^3^Industrial Engineering Department, Yarmouk University, Irbid, Jordan

**Keywords:** psychosocial stress, physical stress, stress, cortisol, heart rate variability

## Abstract

Do physical and psychosocial stressors interact to increase stress in ways not explainable by the stressors alone? A preliminary study compared participants’ stress response while subjected to a physical stressor (reduced or full physical load) and a predetermined social stressor (confronted by calm or aggressive behavior). Salivary cortisol samples measured endocrine stress. Heart rate variability (HRV) and electrodermal activity (EDA) measured autonomic stress. Perceived stress was measured *via* discomfort and stress state surveys. Participants with a heavier load reported increased distress and discomfort. Encountering an aggressive individual increased endocrine stress, distress levels, and perceived discomfort. Higher autonomic stress and discomfort were found in participants with heavier physical load and aggressive individuals. The results suggest a relationship where physical load increases the stressfulness of aggressive behavior in ways not explainable by the effects of the stressors alone. Future research is needed to confirm this investigation’s findings.

## Introduction

Many professions depend greatly on the ability to interact with clients, civilians, patients, students, and customers (Euwema et al., 2004). However, how these professionals handle conflicts with people greatly impacts their ability to do their jobs and may have short-term and long-term physiological, cognitive, social, emotional, and performance effects (Salas et al., 1996; Walker et al., 2014). Conflict situations can include medical professionals interacting with demanding patients (Bakker et al., 2000), teachers confronting student misbehavior and discipline issues (Skaalvik and Skaalvik, 2016), clients becoming assertive when they feel they have been mistreated (Brockmann, 2002), military personnel interacting with hostile civilians (Azari et al., 2010), or police interacting with aggressive civilians (Kop and Euwema, 2001).

Research into the acute effects of stress on physiology have established that repeated or continuous exposure to acute stress over time can have an accumulative biological cost, referred to as allostatic load (Moberg, 2000; McEwen, 2004). Repeated exposure to psychosocial stressors has been shown to cause exhaustion and reflect increased vulnerability for allostatic load in individuals who display impaired habituation to manage the stressor (Kudielka et al., 2006). Further, acute stress in addition to chronic background stress has been shown to reduce physiological recovery time, suggesting that the summation of several acute stressors may also result in allostatic load (Gump and Matthews, 1999). Different sources of acute cognitive workload, social evaluative, and noise stressors have also been shown to have a cumulative physiological effect (Pedrotti et al., 2014). The hypothalamic-pituitary-adrenal (HPA) axis is a key stress response endocrine system that may link both physical and psychosocial stress to the ability to effectively handle conflict. The HPA axis regulates the adaptation to increased demands and enables the individual to maintain allostasis under acute stress.

Acute stressors have been shown to have different physiological and psychological effects based upon task, specific stressors, and combination of stressors. For example, research exploring differences in tasks has shown that social evaluative threat has a strong effect on cortisol reactivity in people performing a mock interview, whereas dancing in front of a crowd has a larger effect (Kirschbaum et al., 1993; Rohleder et al., 2007). This reinforces that task/environment context plays a role in how stressors are perceived (Lazarus and Folkman, 1984). The type of stressor also influences the amount of stress reactivity. Research on the effects from isolated stressors have shown that emotional and noise stressors do not elicit a cortisol response, whereas cognitive stressors and social evaluative threat can elicit cortisol independent of other stressors (Dickerson and Kemeny, 2004). Further, exposure to multiple stressors may have interactive effects. A central feature of the adaptive cost model of stress is that coping may subsequently deplete resources and the capability to meet the demands of multiple stressors (Evans et al., 1996). When assessing the combined effect of acute stressors, cold pressor, and social evaluative threat, participants had higher physiological responses when the two stressors are combined (Minkley et al., 2014). There are also studies that show certain stressors only take a biological toll when coping resources are depleted. For example, noise does not have a clear effect on evoking a stress response but has cortisol reactivity when combined with cognitive performance and threat of loss of money (Allen et al., 2014). Recent experiments have focused on creating more reliable standardized stress tests by combining the social evaluative threat with other stressors in order to induce a potent psychophysiological response (Reinhardt et al., 2012; Smeets et al., 2012; Allen et al., 2014; Finseth et al., 2018). However, there is still ambiguity in the expected magnitude of stress response caused by different tasks, stressor, and stressor combinations.

While social evaluative threat has been investigated in combination with physical stressors like noise and cold, it has not been investigated in combination with a physical load caused by the equipment many professionals are now required to wear, often while interacting with others in stressful encounters. Therefore, the preliminary research described in this paper takes a novel approach by investigating this interaction effect on an individual. Research on carried weight suggests that increasing the weight of carried load will result in increased cardiovascular activity and salivary cortisol levels during a half-hour uphill walk (Paul et al., 2015). Similarly, increasing the weight of law enforcement body armor has been shown to increase heart rate and oxygen uptake during a treadmill test (Myles and Saunders, 1979; Dempsey et al., 2013). Heavy loads can also increase perceived effort, decrease comfort, and deteriorate cognitive performance (Kobus et al., 2010). However, all these studies combine the physical load with intensive cardiovascular activity. In contrast, the study in this paper investigates the effect of physical load with the cognitive stressor of social evaluative threat under more sedentary physical activity. As professionals are required to carry increasingly heavy equipment load while simultaneously interacting with others in socially threatening situations (e.g., soldier, police), understanding the effects of this combination is an important area of study.

Human interactions can strongly affect stress in people across a variety of professions (Saner, 1990; Sharma and Sharma, 2012). For example, soldiers and law enforcement officers have frequent interactions with violent, antisocial, and mistrustful civilians. This social situation, along with the bureaucratic nature of organizations, can negatively affect their psychological health and result in chronic stress (Evans and Coman, 1993; Biggam et al., 1997; He and Lovrich, 2002; Dempsey and Forst, 2013). Teachers have also been shown to experience burnout and exhaustion leading to a dysregulation of stress hormone levels (Bellingrath et al., 2008). Professionals are expected to manage their emotions and display appropriate demeanor even during psychosocial confrontational situations with the public (Rafaeli and Sutton, 1987; Grandey, 2003). Often, the management of displayed emotions requires self-regulation and emotional labor (Grandey, 2000; Goussinsky, 2011). Emotional dissonance occurs when suppressing an emotion and faking the appropriate emotion result in an internal threat to the person’s own identity (Rafaeli and Sutton, 1987; Brotheridge and Lee, 2002; Jansz and Timmers, 2002). Professionals who experience stress from emotional dissonance use more energy and are likely to become emotionally exhausted and unable to regulate their emotions (Wharton, 1993; Morris and Feldman, 1996; Zapf, 2002; Grandey, 2003). Left unaddressed, stressful encounters could result in a break in character, depersonalization, and cynicism (Rafaeli and Sutton, 1987; Aspinwall and Taylor, 1997; Grandey, 2003; Bakker and Heuven, 2006).

Demeanor is a reciprocal relationship, where both parties contribute to the outcome. For instance, disrespect by civilians elicits a strong influence over police behavior and they are more likely to be sanctioned through arrest, citations, or the use of force (Lundman, 1996; Engel et al., 2000). Terrill and Paoline (2007) found that even while acknowledging the officer’s commands or questions, a civilian’s disrespectful statements or actions contributed to the likelihood of being arrested. An officer’s negative demeanor can be a reaction to something a civilian said or did (Klinger, 1996; Dunham and Alpert, 2009). This can be thought of as an emotional contagion where disgruntled civilians can transfer negative emotions to the officer, leading the officer to feel dissatisfied despite an otherwise purposeful encounter (Pugh, 2001; Barsade, 2002). In other cases, nurses’ negative demeanor toward patients could be a result of emotional exhaustion, due to continually suppressing emotional reactions to death, illness, and violence (Bakker and Heuven, 2006). Kop and Euwema (2001) found that rather than the objective situation, it is the degree of emotional exhaustion and depersonalization that determines the use of forceful behavior. This is displayed in a negative interaction pattern, whereby professionals who are cynical and detached behave more forcefully toward others, who subsequently will react in a negative and uncooperative manner which, in turn, will reinforce the negative attitudes of the professionals.

Beyond interpersonal stress, physical challenges may impede emotional control and heighten the stress response. Prolonged physical effort can lead to fatigue and a reduction in physical work (Gawron et al., 2001). Several studies have also supported a relationship between the physical load, fatigue, and cognition (Tomes et al., 2017; Stephenson et al., 2019). The physical effort to carry body armor resulted in diminished higher-level executive processing, which can include attentional control, working memory, and cognitive flexibility (Roberts and Cole, 2013). The fatigue from physical effort can deplete resources needed to regulate mental effort for complex tasks (Staal, 2004). Cognitive resources are also essential for regulating emotional demands (Richards and Gross, 2000). In combination with psychosocial stress, protective body equipment represents physical stress that could influence emotional regulation. The development of protective equipment and non-lethal devices has become a major safety consideration, but also an increasing contributor to physical weight carried by soldiers and officers (Martinez, 2006; Smith et al., 2007; Dempsey et al., 2013). Soldiers routinely wear body armor when patrolling hostile environments, resulting in physiological strain (Roy et al., 2012). Police officers have also been increasingly required to wear body armor, resulting in acute discomfort linked to chronic musculoskeletal disorders (Dempsey et al., 2013; Larsen et al., 2018).

### Motivation and Approach

The serious health impacts that allostatic load presents on human physiological and psychological health, the research of protective equipment load and concurrent research on the emotional labor necessary to manage a social conflict suggest that both acute stresses might result in a biological cost. Do physical stressors interact with psychosocial stressors to significantly increase stress in ways not explainable by the physical and psychosocial stressors alone? The purpose of this initial study is to investigate the effects of equipment load and civilian’s behavioral state during a simulated traffic stop in relation to a person’s Autonomic nervous system (ANS), endocrine, and psychological stress response. It is hypothesized that: (1) increased physical load; (2) experiencing aggressive behavior while interacting with the participant; and (3) their interaction effect will result in elevated ANS and endocrine markers, psychological stress, and discomfort.

## Materials and Methods

### Overview

The protocol for stress induction within this experiment mimics many aspects of the Trier Social Stress Test (TSST; Kirschbaum et al., 1993), which is the most common and reliable laboratory stress induction used in stress reactivity research. First, the TSST is a mock interview where a subject must perform a predefined role while being evaluated on performance. The psychosocial stressor of social evaluative threat has been shown the elicit stress hormone responses and cardiac autonomic responses (Bosch et al., 2009). In order to ground the social stressors in a realistic task, we chose to structure the task such that our participants were trained on a traffic stop procedure and told to act the role of an officer when encountering civilians. The civilian provides social evaluative threat, which can be amplified by the experimental condition of calm vs. aggressive demeanor. Second, the facial expression and attitudes of TSST judges have been shown to change stress reactivity in participants (i.e., neutral face and not paying attention vs. smiling and engaged; Wiemers et al., 2013). Similarly, our civilian confederate displayed facial behavior similar to the TSST protocol. Third, sample sizes of published experiments have shown that TSST reactivity have been recorded for groups of 9–12 participants (Jönsson et al., 2010; Minkel et al., 2014; Montero-López et al., 2016), and other studies that have analyzed cortisol and autonomic measures during laboratory stressor test have used groups of five participants (Simeon et al., 2007; Barker et al., 2010). As a preliminary study, we chose similar sample and group sizes.

### Participants

Study procedures were approved by the Iowa State University Institutional Review Board. For a preliminary investigation, the sample of 20 participants (three females and 17 males) was included in the final preliminary study dataset, with a mean age of 31 (range: 18–44 years). For some dependent variables, technical issues with the equipment resulted in lost data. Individuals from the general population were recruited instead of police officers due to the interference with normal job duties for on-patrol officers. None of the participants reported having previous police training.

While having a sample of real officers would improve the generalizability of the results, the primary goal was to evaluate effects on acute stress response. Compared to the general population, officers receive some awareness training to better prepare them for hostile situations and stress management, but training can range from 30 min to 10 h (Patterson et al., 2014). Stress also varies greatly between police officers depending on a multiple intrapersonal factors including coping ability, cognitive appraisal, past experience, social support, and personality traits (Anshel, 2000; Anderson et al., 2002). Even after receiving police training, officer coping strategies can vary greatly and result in either positive or negative behavioral patterns (Hart et al., 1995). This is evident by large samples of police officers showing elevated heart rate during verbal aggression during routine traffic stops (Tupy, 2014). Trait anxiety and coping styles contribute to the variability in cortisol response and affective reactivity; however, cortisol reactivity may still be significantly higher than control groups without statistically controlling for trait/coping (Villada et al., 2016). Therefore, these studies suggest that acute physiological changes may be generalizable between student and police samples.

### Tasks/Scenarios

Participants played the role of a police officer conducting a traffic stop. The role of the civilian was played by the same confederate from the experimental team in order to ensure consistency in behavior. The participant was seated in a police vehicle parked behind another vehicle.

**Figure 1:**
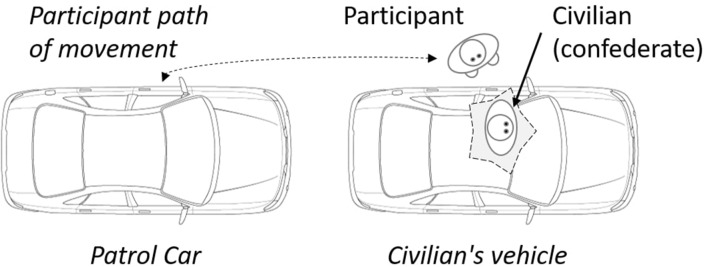
Experimental setup of the two vehicles.

The participant began by radioing dispatch with situation information including the location, license plate number, number of occupants, car color, car type, and any unusual activity. Figure 1 illustrates the experimental setup of the two vehicles. The participant moved between the two vehicles, and the civilian was seated in the vehicle during the scenario.

The participant approached the civilian’s vehicle with caution, confirming the trunk was securely latched, looking for weapons or contraband through the car windows and stopping behind the driver’s side door. The participant requested the civilian to roll down the window, introduced themselves, and asked standard questions: “*Where is your license and registration kept?*” *“Can I have your license and registration?” “Who is the owner of the car?” “What is your address?” “Do you know why you are being stopped?” “Do you have a reason why you got pulled over today?”* and* “Where are you coming from and where are you going?”*

After collecting license and registration, the participant requested the civilian to stay in his vehicle and returned to the police vehicle. The participant radioed dispatch to say they were issuing a ticket and filled out a traffic citation with the information collected. The fine amount and speeding violation were already listed on the citation.

The participant then returned to the civilian vehicle, said, *“You are receiving a ticket,”* and asked the civilian to sign the ticket. The participant returned to the police vehicle and notified dispatch.

### Independent Variables and Experimental Design

A between-subjects 2 (physical load) × 2 (behavioral state) experiment was conducted. Each participant was assigned to one of the four conditions determined by the combination of duty belt (full or reduced) and civilian behavioral state (calm or aggressive).

**Figure 2:**
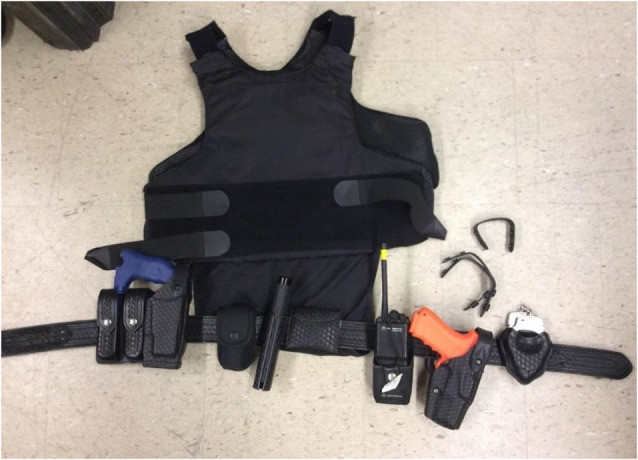
Full-duty belt and body armor.

As illustrated in Figure 2, the full duty belt weighed 13 lbs and included body armor, belt, two magazines, Taser, baton, latex gloves, radio, firearm, and handcuffs. The reduced duty belt weighed 6.3 lbs and included the firearm, belt, and handcuffs.

The behavioral states were defined by actions, attitudes, and responses of the civilian in the scenario. The calm civilian was compliant, answered questions directly, rolled the window down all the way, and did not hesitate to sign the citation. The aggressive civilian was argumentative (“*Do you really have nothing better to do today than pull me over?*”), not compliant, hostile, loud, avoided answering questions, made extravagant hand gestures, and resisted signing the citation.

### Dependent Variable Measures

Both psychological and physiological indices of stress were measured. The dependent variables are summarized in Table 1.

**Table 1:** Description of dependent variable metrics, units, and frequencies.

Dependent variable	Metric	Components	Association	Measurement frequency
Autonomic stress response	Heart rate variability (HRV)	HR	Cardiac activity	Before trial, 30-s interval throughout trial
		HF, HF n.u.	Parasympathetic (i.e., vagal activity)
		LF, LF n.u.	Sympathetic and parasympathetic
		LF/HF ratio	Sympathovagal balance
Autonomic stress response	Electrodermal activity (EDA)	Skin conductance level (SCL)	Sympathetic (tonic)	Before trial, 60-s interval throughout trial
		Number of skin conductance responses (NSCR/min)	Sympathetic (phasic)
Endocrine stress response	Salivary cortisol		Hypothalamic-pituitary-adrenal (HPA) axis	Before trial, 5 min after trial, 15 min after trial
Discomfort	Discomfort survey	Likert scale		Before trial, After trial
Psychological stress response	Short Stress State Questionnaire (SSSQ)	Likert scale		Before trial, after trial
Psychological stress response	Informal interview	Open-ended questions		After trail

#### Autonomic Stress Response

ANS arousal has been shown to be associated with social evaluative threat (Bosch et al., 2009) as well as load carriage (Ribeiro et al., 2014). ANS responses to stress were examined with two measures: heart rate variability (HRV) and electrodermal activity (EDA). HRV is the change in the time interval between consecutive heartbeats, which can be obtained by measuring ANS frequency indices that reflect arousal or relaxation. Two overlapping branches in the ANS, sympathetic nervous system branch and parasympathetic nervous system branch, determine the arousal or restorative functions. While interplay between the two branches determines the overall cardiovascular system stress response, each ANS branch can experience activation or withdrawal resulting in prevailing effects by the other branch. The parasympathetic nervous system predominates the resting state by slowing down the heart rate, whereas the sympathetic nervous system is the principal way to speed up the heart rate from the intrinsic rate of the heart’s biological pacemaker. Sympathovagal balance is an index of the relative amount of sympathetic activity relative to parasympathetic activity of the ANS. Lower levels of parasympathetic activity, higher levels of sympathetic activity, or higher levels of sympathovagal balance reflect the activation of the individual’s physiological stress response. Electrocardiogram (ECG, modified CS_5_ lead configuration; Malik, 1996) recorded HRV, sampled using Thought Technologies ProComp Infiniti (2,048 Hz) recordings. Participants were asked to remain seated and quiet for 10 min, while baseline data was collected. Spectral analysis of HRV was performed using the Matlab-based toolbox Kubios HRV (V2.2; Niskanen et al., 2004). The Kubios software used artifact correction and linear trend removal of low-frequency trend components (frequencies below 0.04 Hz) from the RR interval series. Spectral density analysis of the HRV parsed the data into a high-frequency (HF; 0.15–0.4 Hz) band reflecting parasympathetic control of the heart *via* the vagus nerve, and a low-frequency (LF; 0.04–0.15 Hz) band reflecting sympathetic activity with vagal modulation. The very low-frequency (VLF; <0.04 Hz) band was not included in this study because it is unreliable for short-term recordings (<5 min; Malik, 1996). The LF and HF components were normalized to their total power in order to remove influences of VLF (e.g., HF n.u. = HF/(HF + LF) × 100). LF/HF ratio was calculated to assess sympathovagal balance. The HRV frequency bands for each participant were calculated in 60-s intervals over the duration of the trial.

EDA measures changes in electrical conductivity in the skin due to aroused production of sweat by activation of the ANS. Increased arousal during stress will elicit higher EDA. EDA can be parsed into slower tonic-level and faster-changing phasic-level components. Skin conductance level (SCL) is a measure of tonic EDA and reflects the general changes in autonomic activity. Skin conductance response (SCR) is discrete, short, phasic fluctuations that reflect higher frequency variability of the signal to immediate stimuli (Figner and Murphy, 2011). EDA was sampled with the ProComp Infiniti (256 Hz) and placed on the intermediate phalanges on the index and middle fingers of the non-dominant hand for least intrusion during the study. The skin conductance data were analyzed with the Matlab-based toolbox Ledalab (V3.4.9; Benedek and Kaernbach, 2010). Skin conductance data was downsampled to 8 Hz. Subsequent removal of artifact-afflicted trials and data smoothing were carried out using Ledalab. Continuous decomposition analysis (CDA) with optimized goodness of fit decomposed the data into continuous phasic and tonic components. For each experimental condition, the number of skin conductance responses (NSCR) was extracted and calculated per minute. The threshold for detecting significant skin conductance responses was 0.01 μs. The tonic SCL was obtained from the CDA at 30-s intervals over the duration of the trial and then used to calculate the relative difference from baseline.

#### Endocrine Stress Response

Cortisol, is an indicator of HPA axis response to a social evaluative stressor like peer aggression (Knack et al., 2011). Cortisol is a stress hormone measured by free cortisol concentrations through salivary samples. Cortisol can indicate adrenocortical activity and has a high predictive value of psychosocial stress (Foley and Kirschbaum, 2010). Cortisol peak levels generally occur 10–30 min after a stressor (Kirschbaum et al., 1993). To measure the cortisol response curve, three salivary samples were obtained to over the expected response curve: baseline, cortisol onset beginning 5 min after experimental scenario, and expected peak cortisol level at 15 min after the experimental scenario. Before each sample collection, the participant filled out a Saliva Collection Survey to account for variation between individuals. Before the first sample collection, the survey asked questions regarding awakening, time of collection, medication use, mood, sleep, daily hassles or uplifts, and other control variables for salivary hormones. For all additional samples, the survey only asked about participant mood in that particular moment (Shirtcliff et al., 2005). Each participant was in the laboratory for at least a 30-min period before any stress measurements were recorded to minimize HPA axis stress response caused by arrival to the laboratory (Shirtcliff et al., 2014).

The samples were obtained from participants using a passive-drool method directly into a cryovial tube. Samples were stored at −80°C. The samples were thawed to room temperature and spun at 3,000 rpm for 10 min to obtain 0.5–1.0 ml clear saliva. Salivary cortisol concentrations were determined by commercially available enzyme immunoassay (Salimetrics, State College, PA, USA). Original samples were assayed in duplicate. Intra-assay coefficients of variation (CVs) averaged 11.5%, and the inter-assay CV averaged across low and high controls was 6.16%. Samples with CVs >10 were assayed again. Cortisol means (*M* = 0.16 μg/dl, SD = 0.22 μg/dl), and baseline cortisol means (*M* = 0.13 μg/dl, SD = 0.14 μg/dl) were in normal ranges.

#### Discomfort

A postural discomfort survey (Corlett and Bishop, 1976) asked the participant to determine the discomfort level on a 1- to 5-point scale for various parts of the body: eyes, neck, head, upper back, lower back, upper arm, elbow, hip, and thigh. The discomfort was scored as percentage from pre- to post-trial for each body segment.

#### Psychological Stress Response

The Short Stress State Questionnaire (SSSQ) assessed the subjective states pre- and post-trial to measure three state factors: task engagement, distress, and worry (Helton, 2004). Engagement refers to qualities of energetic arousal, motivation, and concentration. Distress is defined by feelings of tense arousal, hedonic tone, and confidence control. Worry relates to self-focus, self-esteem, and cognitive interference (Matthews et al., 1999). The stress state acts as a mediator between the stressor and cognition or information processing, whereby the three aspects represent components of conscious experience during person–task–environment transactions (Helton and Näswall, 2015). The three-factor SSSQ scale scores for pre- and post-trial were calculated for each participant. The factor scores from both pre- and post-trial are standardized against normative means and standard deviation values from a large sample of British participants obtained by Matthews et al. (2002) and standardized using methods by Helton (2004) and Helton et al. (2009). Change scores were calculated for each factor using the formula, *z = (standardized post-score − standardized pre-score)*, which has been used in previous studies (Helton and Näswall, 2015). Normalization of the SSSQ scores has been the standard convention used by the SSSQ authors (Matthews et al., 2002; Helton, 2004). Change scores allow for convenient comparison across multiple samples and studies (Matthews et al., 2002).

#### Procedure

**Figure 3:**
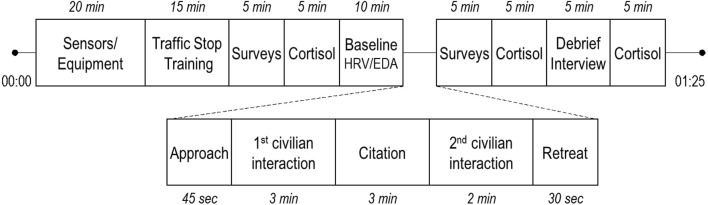
Experiment approximated timeline.

The experiment (see Figure 3) was conducted in a large, temperature-controlled garage workspace. A demographic survey was administered, and height and body weight measurements were collected. Participants were equipped with the law enforcement gear (either full or reduced) and ECG and EDA sensors.

Prior to beginning the scenario, all participants were given a basic training on how to conduct a traffic stop. This training was directly created from the Iowa Law Enforcement Academy (ILEA). This training focused on Location (awareness of the situations), Approach (how to safely approach the vehicle), Awareness (actions taken to inventory the situation prior to and during initial contact, including return to patrol vehicle), Re-approach (actions taken during second contact, including actions taken on return to patrol vehicle), and Release (the end of encounter and official return to available status). Participants were trained on how to properly fill out a citation sheet and standard civilian interaction protocols. While still seated, participants completed a discomfort survey, SSSQ, Saliva Collection survey, and gave their first saliva sample. The participant then remained seated for 10 min to collect baseline ECG and EDA data.

Next, participants conducted the scenario in a simulated traffic stop. The same researcher role-played the civilian for all participants. The scenario finished when the participant told dispatch they issued the ticket. Following the interaction, another discomfort survey and SSSQ were administered. Five minutes after the completion of the interaction, a second saliva sample was collected. A debrief interview was conducted while still seated. Fifteen minutes after the end of the scenario, the third saliva sample was collected. Participants then removed sensors and gear. A short debriefing requested that participants refrain from discussing the experiment.

#### Data Analysis Plan

Linear mixed models (LLM) with first-order autoregressive AR(1) covariance structure determined the statistical significance of independent variables on HRV and EDA. Random effect from participant sampling was used for the HRV and EDA analysis. For EDA, the tonic SCL relative difference from baseline and phasic NSCR was normally distributed. All HRV, HR, and cortisol concentrations were winsorized to three standard deviations. The LF/HF, LF, and HF variables had a moderate positive skew and were log(x) transformed. The analysis for the stress state questionnaire (SSSQ) was a two-way ANOVA using the factors behavioral state and physical load. The discomfort was normalized due to positive skew and analyzed using Mann–Whitney U. Results were considered significantly for *p* ≤ 0.05 and marginally significant for 0.05 < *p* ≤ 0.1 (Gelman, 2013).

A ln(Cort) + 5 transformation was applied to normalize the cortisol data. Hierarchical linear modeling (HLM) growth curve analyses (Raudenbush and Bryk, 2002; Singer and Willett, 2003; HLM 7.01) characterized changes in cortisol concentrations for intra-individual differences in samples (Meyer et al., 2015). Three samples from each individual shaped the hormonal response curve with respect to time. In the HLM analysis, cortisol was the predicted variable and the time variable (time since beginning of experiment) were the Level 1 predictors. The behavioral state and physical load were used as Level 2 predictors.

Effect size for the standardized mean difference was calculated for the fixed effects and interaction effect. The influence of upward bias from sample sizes less than 20 (or less than 10 in each group) was corrected by computing the d_unbiased_, also called Hedges’*d* (Hedges and Olkin, 1985). Hedges’*d* effect size guidelines are adopted as small for 0.2 < *d* < 0.5, medium for 0.5 < *d* < 0.8, and large for *d* > 0.8 (Cohen, 1988). Effect sizes for the Mann–Whitney *U* tests were calculated with normal approximation z to r. Cohen’s guidelines for Pearson correlation *r* score effect size are adopted as small for 0.1 < *r* < 0.3, medium for 0.3 < *r* < 0.5, and large for *r* > 0.5 (Fritz et al., 2012).

## Results

### Heart Rate Variability

Physiological data is presented in Table 2. Only 16 participants’ data were used due to data loss from faulty equipment. This resulted in unequal participant group numbers of Reduced/Calm (*n* = 4), Full/Calm (*n* = 5), Reduced/Aggressive (*n* = 4), and Full/Aggressive (*n* = 3).

**Table 2:** Descriptive statistics for Linear Mixed Models (LMM) measures of heart rate variability (HRV), listing means (SE).

Metric	Reduced load		Full load	
	Calm	Aggressive	Calm	Aggressive
log(LF) (ms^2^)	2.89 (0.19)	3.04 (0.19)	2.64 (0.19)	2.99 (0.18)
log(HF) (ms^2^)	2.48 (0.27)	2.70 (0.27)	2.35 (0.27)	2.24 (0.26)
LF n.u.	70.2 (5.10)	68.7 (4.58)	62.0 (4.76)	81.3 (4.18)
HF n.u.	29.7 (5.09)	31.2 (4.57)	37.9 (4.76)	18.7 (4.18)
log (LF/HF)	0.43 (0.12)	0.37 (0.11)	0.27 (0.11)	0.74 (0.095)
HR (BPM)	94.6 (7.72)	95.0 (7.57)	105 (7.64)	89.8 (7.45)

The main effects of physical load and behavioral state were not significant on the log(LF) component. The interaction effect was not significant. The main effects of physical load and behavioral state were not significant on the log(HF) component. The interaction effect was not significant.

**Figure 4:**
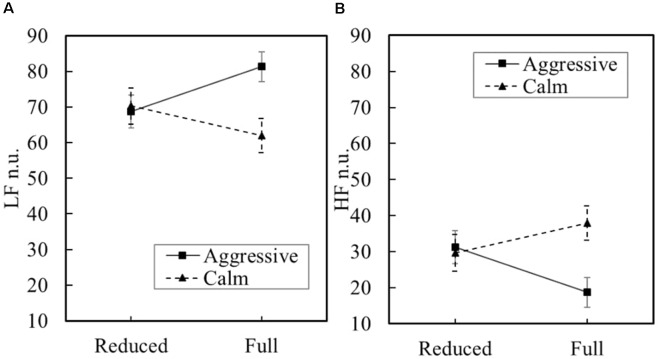
Means and standard error for normalized low-frequency (LF; **A**) and normalized high-frequency (HF) bands **(B)**. Measured at 30-s intervals throughout trial.

The main effect of physical load was not significant on the normalized LF (Figure 4A). The main effect of behavioral state on the normalized LF was marginally significantly greater for the aggressive civilian (*M* = 75.0, *SE* = 3.10) than for the calm civilian (*M* = 66.1, *SE* = 3.49), *F*_(1,12.68)_ = 3.62, *p* = 0.08, *d* = 0.90. The interaction effect was significant, *F*_(1,12.68)_ = 4.97, *p* = 0.045, *d* = 1.05. The main effect of physical load was not significant. The main effect of behavioral state on the normalized HF was marginally significantly greater for the calm civilian (*M* = 33.8, *SE* = 3.48) than for the aggressive civilian (*M* = 24.9, *SE* = 3.10), yielded *F*_(1,12.68)_ = 3.61, *p* = 0.08, *d* = 0.90 (Figure 4B). The interaction effect was significant, *F*_(1,12.68)_ = 4.97, *p* = 0.044, *d* = 1.05, indicating that the behavioral state effect was greater in the full condition than in the reduced condition.

**Figure 5:**
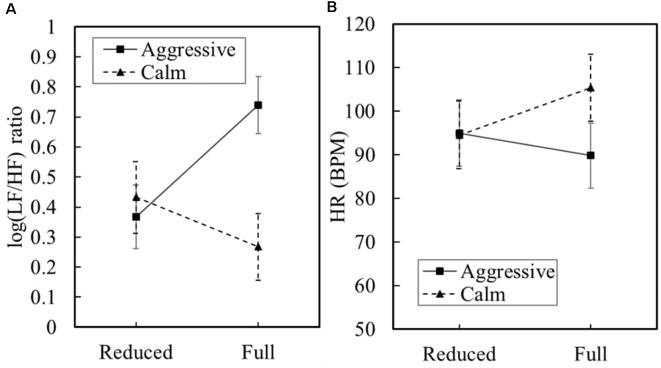
Means and standard error for LF/HF ratio **(A)** and heart rate beats per minute (BPM; **B**). Measured at 30-s intervals throughout trial.

The main effect of physical load was not significant on the LF/HF (Figure 5A). The main effect of behavioral state on the LF/HF ratio was marginally significantly greater for the aggressive civilian (*M* = 0.55, *SE* = 0.071) than for the calm civilian (*M* = 0.35, *SE* = 0.082), yielded *F*_(1,12.68)_ = 3.55, *p* = 0.082, *d* = 0.89. The interaction effect was significant, *F*_(1,12.68)_ = 6.18, *p* = 0.027, *d* = 1.18, indicating that the behavioral state effect was greater in the full condition than in the reduced condition. The main effect of physical load was not significant on HR (Figure 5B). The main effect of behavioral state was not significant. The interaction effect was not significant.

### Electrodermal Activity

Only 16 participants’ data were used for the analysis in similar groups as the HRV. The main effects of physical load and behavioral state were not significant on SCL. The interaction effect was not significant. The main effect of physical load and behavioral state was not significant on the NSCR per minute. The interaction effect was not significant.

### Endocrine Stress Response

Only 18 participants’ data were used in this analysis. This resulted in differing participant group numbers of Reduced/Calm (*n* = 5), Full/Calm (*n* = 5), Reduced/Aggressive (*n* = 4), and Full/Aggressive (*n* = 4). An initial base model was calculated to determine if changes in the stress hormone were due to inter-individual variability vs. intra-individual variability or moment-to-moment variance in cortisol. Differences between individuals was attributed to 71% of the variance and momentary fluctuations within an individual was attributed to 29% of the variance, $\chi_{\left(17\right)}^{2}$ = 139, *p* < 0.001. The resulting intra-class correlation coefficient indicates that HLM is an appropriate technique. To categorize reactivity status in all participants, 67% (*n* = 12) showed increased cortisol in response to the traffic stop. Of the 33% (*n* = 6) non-responders who showed a constant or decreasing hormonal slope, three out of the six non-responders were in the calm/reduced experiment condition.

**Figure 6:**
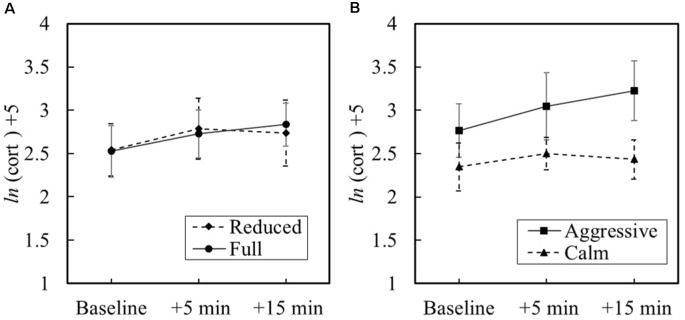
Cortisol response and standard error for **(A)** the physical load and **(B)** the behavior.

The main effect of physical load was not significant on the peak cortisol concentration (Figure 6A). For the peak cortisol response occurring 15 min after the simulation, as illustrated in Figure 6B, the growth curve slope due to the civilian’s behavioral state was marginally significant, *γ* = 0.20, *t*_(16)_ = 2.00, *p* = 0.063, indicating increased reactivity during peak concentration for the aggressive civilian state. The main effects and interaction were not significant before the simulated traffic stop (i.e., baseline). No main effects or interaction effects were found for the sample growth curve.

### Discomfort

Only 17 participants’ data were used in this analysis. This resulted in differing participant group numbers of Reduced/Calm (*n* = 4), Full/Calm (*n* = 5), Reduced/Aggressive (*n* = 4), and Full/Aggressive (*n* = 4). The discomfort data was normalized to correct for positive skew.

The main effect of physical load on hip was marginally significant (*U* = 18, *p* = 0.098, *r* = −0.97), where the full belt (*Mdn* = 11.0) was greater than the reduced belt (*Mdn* = 6.75). The main effect of physical load was not significant on any other body parts. The main effect of behavior was not significant on any body parts.

### Psychological Stress Response

**Figure 7:**
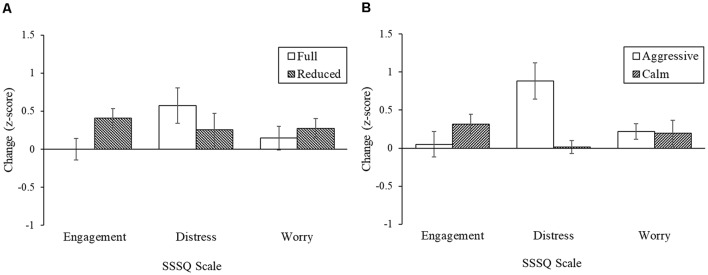
State changes for the Short Stress State Questionnaire (SSSQ) factors for the **(A)** physical load and **(B)** civilian behavior. Error bars are the standard errors.

Only 17 participants’ data were used in this analysis. This resulted in differing participant group numbers of Reduced/Calm (*n* = 4), Full/Calm (*n* = 5), Reduced/Aggressive (*n* = 4), and Full/Aggressive (*n* = 4). The data was normally distributed. The SSSQ results for task engagement, distress, and worry are presented for physical load (Figure 7A) and behavioral state (Figure 7B).

The main effect of physical load was significant for engagement, *F*_(1,13)_ = 5.41, *p* = 0.037, *d* = 1.28, where engagement increased as load decreased. The main effect of behavioral state and the interaction effect were not significant for engagement. The main effect of physical load was not significant for distress. The main effect of behavioral state was significant for distress, *F*_(1,13)_ = 14.3, *p* = 0.002, *d* = 2.08, where an increase in civilian aggression increased participant distress. The interaction effect was not significant. The main effects of physical load and behavioral state and the interaction effect were not significant for worry.

## Discussion

The first hypothesis of an effect of physical load was partially supported by subjective distress and discomfort. The physiological markers did not show significant increase in stress due to increased load. There was no change in peak cortisol levels due to load alone. The heavier physical load of the equipment increased psychological distress and marginally resulted in physical discomfort in the hips. Studies have shown that physical loads at 15% of body weight cause significant muscular stress, with loads surpassing 30% of body weight significantly increasing perceived exertion and fatigue (Quesada et al., 1996). The progressive increases in carried load can result in muscle discomfort and psychological distress (Johnson et al., 1995). The past research supports the current findings that physical load can contribute to distress and discomfort.

Discomfort may also be attributed to the position of the duty belt and geometry of the worn equipment. Physical loading on concentrated regions can lead to discomfort through factors such as joint angle, tissue pressure, muscle contractions, blood pooling and circulation blockage (Helander and Zhang, 1997). These discomforts can result in the perception of pain, tiredness, soreness, and numbness. In the current study, some participants reported discomfort in their hips and that they were annoyed with the gear while sitting and getting out of the car. Other studies have reported similar associations between police belts and elevated discomfort in standard vehicle seats (Czarnecki and Janowitz, 2003; Filtness et al., 2014). A study investigating discomfort from police duty and driver seats found increased discomfort for a full-duty belt configuration compared to a reduced duty belt configuration (Holmes et al., 2013). Together, this research supports the current experimental finding that there is a difference in discomfort and distress when comparing a full and reduced duty belt configuration.

The second hypothesis of an effect of civilian behavior was partially supported by increases in HRV, cortisol peak level reactivity, and SSSQ distress. Psychophysiological axes are interrelated and should show similar responses across dependent variables, even if statistical power and significance are achieved or not. These effects are interpreted to be true positives because the results are consistent with psychological and physiological stress anatomical pathways and past experimental research. When assessing the autonomic activity through HRV in combination with EDA, results indicated parasympathetic modulation as the primary mediator for the stress response. The HF component of HRV reflects parasympathetic nervous system effects, whereas the LF component reflects both parasympathetic and sympathetic cardiac modulation. Therefore, the LF/HF ratio is taken as a measure of sympatho-vagal balance. When compared in unison, the aggressive behavior of the civilian was found to marginally increase the autonomic stress response as reflected by a trend in parasympathetic withdrawal.

When assessing the endocrine stress response, higher reactivity during peak cortisol levels only partially supported the second hypothesis and was marginally associated with the behavior displayed by the civilian: higher cortisol levels resulted from aggressive behavior vs. a calm civilian. Psychologically, the aggressive behavior of the civilian led to an increase in distress levels of the participants. Participants reported, “I felt stressed when the civilian was giving excuses and refusing to sign the ticket.” Loss of control is a common contributor to a distress response (Funke et al., 2007; Matthews et al., 2013). Uncontrollability can refer to delayed recovery when demand exceeds resources (Koolhaas et al., 2011). This suggests that the civilian’s social aggression resulted in a perceived loss in control demanding enough to enact multiple psychological systems. The peripheral cortisol response occurred in tandem with autonomic stress response to suppress any distress and further enable the body to recover. Further, participants also reported, “[The] civilian affected my reaction heavily, he put more stress to the situation and I added to the stress too.” Literature has shown cortisol to be associated with aggression in retaliation to a social provocation (Van Bokhoven et al., 2005). During these hostile interactions, HPA axis activation in the receiver causes enhanced aggressive behavior, which then, in turn, further activates the HPA axis (Kruk et al., 2004; Böhnke et al., 2010). This negative interaction pattern was previously documented for law enforcement (Kop and Euwema, 2001).

The third hypothesis was partially supported by increases in HRV. The large LF/HF ratio suggests that wearing full equipment during an aggressive encounter induces parasympathetic withdrawal, consequently losing the restorative abilities and making the individual more stressed (Shaffer et al., 2014). Results indicate stress levels were less when wearing reduced equipment during the same aggressive encounter. However, in a calm situation, differing equipment alone did not influence stress level. This suggests a relationship where increased physical load increases the stressfulness of aggressive behavior more than can be attributed to stress of the behavioral state of the civilian alone. The interpretation of HRV results is supported by studies finding that short lasting exposure to psychosocial stressors can cause parasympathetic withdrawal with an unchanged sympathetic activity to be responsible for increase in LF/HF ratio (Hjortskov et al., 2004). When assessing the sympathetic activation by method of EDA, the absence of group difference in SCL and SCR vindicates sympathetic response and reaffirms the HRV stress response is primarily from parasympathetic withdrawal.

Parasympathetic function is critical to emotional regulation during face-to-face interactions, especially in situations of emotional dissonance and expressive suppression (Butler et al., 2006). Conducting a traffic stop on an aggressive civilian required the display of respectful demeanor, consequently eliciting a stress response from the management of negative emotions. However, individuals with reduced physical load during aggressive encounters displayed higher levels of parasympathetic modulation, associated with better control over negative emotion during ongoing stressful situations (Thayer et al., 2009). This suggests that higher physical loads may consume resources needed to regulate emotion. Reduction in carried loads may free up cognitive resources leading to better emotional control, more favorable interactions between enforcement officers and civilians, and lower the prevalence of emotional exhaustion among officers. Further, emotion regulation can consume cognitive resources needed for tasks (Spangler et al., 2015). Officers are expected to maintain a neutral demeanor and more effort is needed to regulate their dissonant feelings (Huang and Dai, 2010). Therefore, higher load carriage could result in a diminished cognitive task performance.

While the cortisol growth curve was not associated to the behavioral state and physical load, this is presumably because: (a) the second and third salivary samples were collected too early to measure cortisol onset and peak; or (b) the physical load was insufficient to warrant an HPA response. The positive rate of change for the aggressive behavior condition suggests that the fourth cortisol sample taken 30 min after the end of the experiment scenario may have more accurately modeled the peak cortisol levels. However, even though the behavior groups showed a difference in parasympathetic activity and cortisol reactivity, another explanation is that the physical load might have been a minor stressor that failed to demonstrate an HPA axis response in the time of a single traffic stop. When an external stressor is present, stress is regulated across multiple systems that are heavily influenced by length and severity of the stressor. In the short time, the individual may have been able to cope prior to initiating the cortisol stress response (Shirtcliff et al., 2014). Longer-term effects of physical carried load on officers conducting routine traffic stops may have different implications.

There were several limitations to the study. As a preliminary study, we constrained the sample size and recruited individuals from the general population. Given the variance of police officer stress management programs and intrapersonal factors suggest that acute physiological changes may be generalizable between student and police samples. However, the results may be underpowered and should be interpreted with caution until an investigation can be conducted on a larger sample or a specific population, such as police officers.

Although a full and reduced belt were used to determine the induced stress of the participant, another limitation is that it is hard to distinguish if the stress was a direct result of the weight or belt protruding uncomfortably into the lower back/hips. The reduced belt had gear removed from the posterior region, unlike the full belt. It has been noted in previous studies, that when seated, the participants mentioned considerable discomfort due to the equipment pressing between the seat and their spine (Holmes et al., 2013). This protrusion could have induced stress for the full belt subjects that are unaccountable for the reduced belt subjects. While the physical load in this study was within carrying capacity (Fergenbaum, 2007), adults who are unhabituated with equipment may perceive feelings of discomfort and may respond differently based on physiological conditioning (Roberts and Cole, 2013). Since discomfort is a multi-dimension concept, the metrics used in this study are unable to discern the influence of discomfort vs. load. Therefore, any effect of the belt in the current study may not be attributed to weight alone. However, the experimental full-duty belt and body armor accurately represents law enforcement equipment, suggesting that more research is needed to ascertain whether reduced equipment load or redesigned equipment geometry can minimize the resultant stress response.

## Conclusion

The purpose of this study was to investigate the physical and psychosocial stresses and their interaction effect on an individual. Physical load used in this experiment only contributed to autonomic stress when also subject to an aggressive social interaction, whereas, the aggressive behavior from another person was able to elicit discomfort, autonomic, hormonal, and psychological changes. The simultaneous effects of increasing equipment load and hostile personal interactions consequentially increases stress in ways not attributable to the individual effects of the stressors alone. These preliminary finding suggest that further research is needed into how acute stressors accumulate for professionals who have social interactions. Further, when multiple acute situations lead to chronic conditions, HPA dysfunction from prolonged allostatic load is a major contributor to depression, poorer decision making, and burnout (McEwen, 2004; Bakker and Heuven, 2006). With the increasing amount (and associated load) of mandatory equipment for police officers, soldiers, and other law enforcement personnel, unaltered working conditions could increase prevalence of chronic pathological illness and higher rates of civilian–officer violence. Therefore, how stressors accumulate and effect an individual warrants further study.

## Author's Note

Student authors have been listed alphabetically in the author list.

## Data Availability Statement

The datasets generated for this study are available on request to the corresponding author.

## Ethics Statement

The studies involving human participants were reviewed and approved by Iowa State University Institutional Review Board. The patients/participants provided their written informed consent to participate in this study.

## Author Contributions

All authors contributed to the conception and design of the study. EA, ZE, TF, AM, and ZW executed the study and collected the data and wrote sections of the original manuscript. EA and TF were primarily responsible for the statistical analysis, with oversight from MD and RS. TF led the revision process, with substantial contributions from EA and AM. MD and RS provided editorial oversight throughout both the writing and revision processes. All authors approved the submitted version.

## Conflict of Interest

The authors declare that the research was conducted in the absence of any commercial or financial relationships that could be construed as a potential conflict of interest.
